# Surgical coronary angioplasty of both coronary ostia after chest radiotherapy. Is it good alternative to conventional coronary bypass surgery?

**DOI:** 10.1177/02676591231221707

**Published:** 2023-12-08

**Authors:** Arslan Mamedov, Eglė Rumbinaitė, Dainius Karčiauskas, Gabrielė Jakuškaitė, Audronė Veikutienė, Povilas Jakuška, Rimantas Benetis

**Affiliations:** 1 Department of Cardiac, Thoracic and Vascular Sugery, Medical Academy, 230647 Lithuanian University of Health Sciences, Kaunas, Lithuania; 2 Departament of Cardiology, Medical Academy, 230647 Lithuanian University of Health Sciences, Kaunas, Lithuania; 3 Institute of Cardiology, Medical Academy, 230647 Lithuanian University of Health Sciences, Kaunas, Lithuania

**Keywords:** coronary ostial stenosis, aortic valve stenosis, coronary artery disease, surgical angioplasty, myocardial revascularisation

## Abstract

**Introduction:**

Isolated coronary ostial stenosis of both ostia is a rare, potentially life-threatening condition, occurring in 0.1%–0.2% of patients undergoing coronary angiography.

**Case report:**

We present a case of a 69-year-old woman with a past medical history of breast cancer, who had been treated with radiotherapy, which most likely caused significant stenosis of both coronary ostia and likely accelerated aortic stenosis. Surgical angioplasty with autopericardium patch reconstruction of the left main coronary artery and right coronary arteries due to proximal stenotic disease was performed instead of venous or arterial bypasses with concomitant aortic valve replacement. The postoperative course was uneventful. There were no cardiovascular events 5 years after operation, and the patient remained free of any symptoms.

**Conclusions:**

Surgical coronary angioplasty offers an alternative to conventional coronary artery bypass grafting in isolated coronary ostial lesions and is advantageous in restoring more physiological myocardial perfusion, especially in those cases when conduits are suspected to be fibrotic, scarred or stenosed after radiation therapy or if there is the need to preserve conduits for future myocardial revascularisation in young patients.

## Introduction

Surgical angioplasty (SA) offers an alternative to conventional coronary artery bypass grafting (CABG) in isolated coronary ostial lesions and was first described by Effler et al.^1^ and Sabiston et al.^2^ as a technique whereby coronary reconstruction could be achieved using an autologous patch graft.^3^ We have successfully used SA with concomitant aortic valve replacement (AVR) in one patient after cancer treatment (breast radiotherapy) with suboptimal quality arterial and venous conduits, and significant stenosis of both coronary ostia. Written informed consent was obtained from the patient for publication and photos associated with this case report.

## Case report

**Figure 1.**
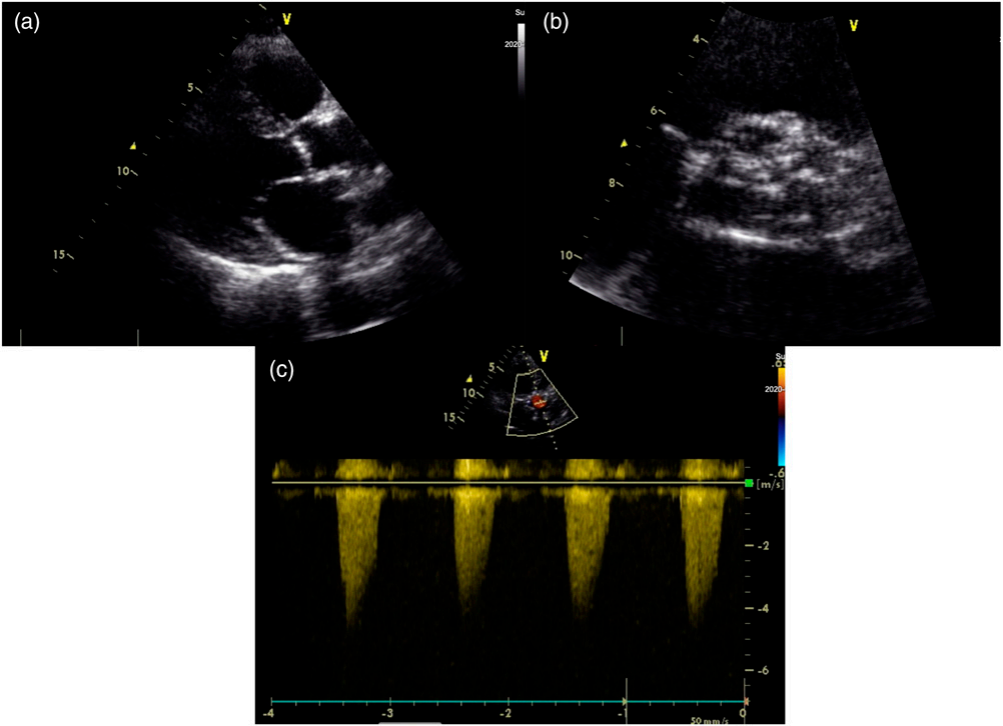
Preoperative echocardiography findings: (a) parasternal long-axis view showing reduced aortic valve leaflets motion, (b) parasternal short-axis view showing significant calcification of aortic valve leaflets, (c) peak aortic jet velocity measured by CW doppler.

**Figure 2.**
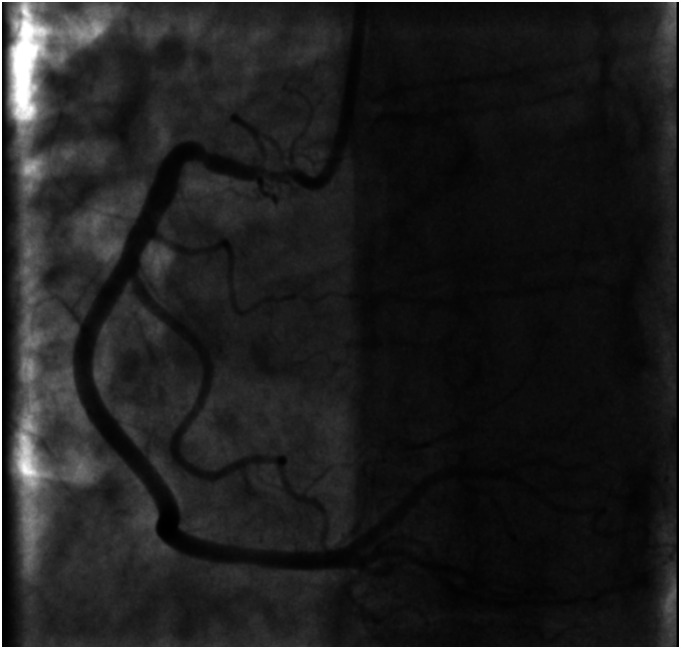
Invasive coronary angiography: right coronary artery ostium stenosis.

**Figure 3.**
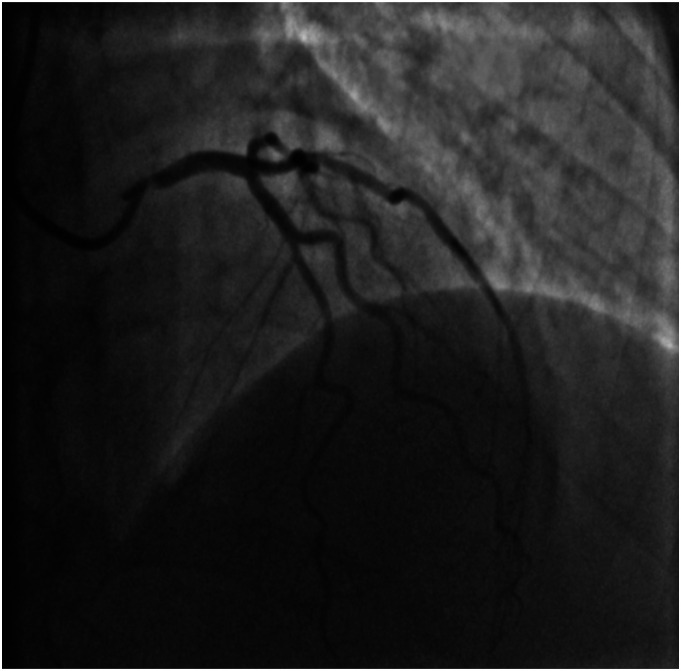
Invasive coronary angiography: left coronary artery ostium stenosis.

A 69-year-old female, with a past medical history of breast cancer (surgical treatment and radiotherapy 10 years ago) was referred to the Neurosurgery Department for cerebral angiography which showed a high-grade stenosis in the proximal left subclavian artery that was successfully corrected (angioplasty and stenting via radial approach). Due to a holosystolic murmur, she was consulted by a cardiologist and severe aortic valve (AV) stenosis was detected. Transthoracic echocardiography (TTE) showed significant AV calcification and reduced mobility of all three leaflets (mean gradient 57 mmHg, AV area (AVA) 0.49 cm^2^, AVA index 0.27 cm^2^) with less than moderate aortic regurgitation (Figure 1). The left ventricle (LV) was hypertrophied and non-dilated with preserved systolic function. Invasive angiography revealed localised proximal 90% right coronary artery (RCA) stenosis (Figure 2) and 75% left main coronary artery (LMCA) ostial stenosis (Figure 3). The heart team decision was surgical myocardial revascularisation and AVR.

**Figure 4.**
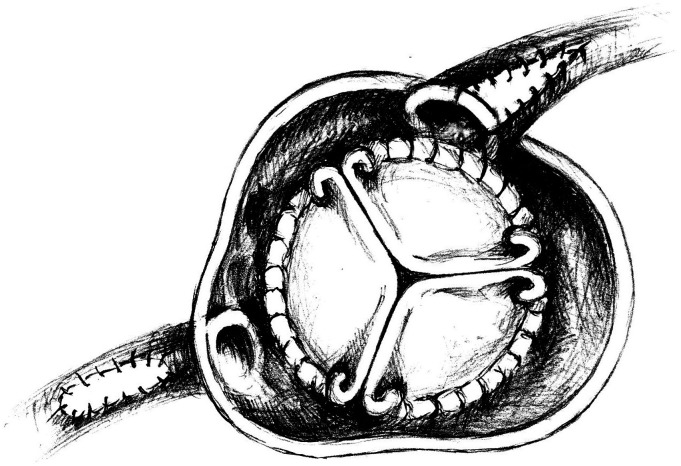
Left main coronary artery and proximal segment of right coronary artery surgical angioplasty using autopericardium patch; aortic valve replacement using tissue valve.

The patient was operated on. After median sternotomy, severe extensive adhesions were noticed mostly around left ventricle and were carefully released to achieve cardiopulmonary bypass (via ascending aorta and the right atrium). The ascending aorta was then cross-clamped and the crystalloid cardioplegia was delivered into the aortic root for diastolic cardiac arrest. Due to dense adhesions and shortage of conduits (extensive varicose veins of lower extremities and abnormal Allen test of the radial artery), after aortotomy was completed 1 cm above the sinotubular junction, the LMCA ostium opening was approached from the aortic valve side and split into the left main stem to directly reach the entire stenotic lesion. The adhesion-free autopericardium patch (right ventricle outflow tract) was taken and sewn (approximately 3 mm wide and 2 cm long) to form a new LMCA ostium. Proximal RCA angioplasty was performed starting with direct incision into the proximal RCA and fresh autopericardium material (3 cm long and 3 mm wide) was also used for reconstruction (Figure 4). After completion of the SA, the AV was replaced using tissue valve (21 mm Trifecta valve). Echocardiography showed normal AV prosthesis function and preserved function of both ventricles. The aortic cross-clamp time was 83 min, and the cardiopulmonary bypass (CPB) time was 115 min.

The patient’s postoperative course was uneventful and she was discharged to a rehabilitation clinic on the 9th postoperative day. The administration of coumadin (warfarin) and one antiplatelet agent (aspirin) was recommended for 3 months, followed by continuing treatment with a dual antiplatelet therapy (aspirin and clopidogrel) for up to 6 months, with one antiplatelet agent being used for life-long therapy. Five years after the operation, the patient's condition remained stable with no major adverse cardiac events and the stress exercise test showed no signs of ischemia.

## Discussion

We present a successful case for both LMCA ostial and RCA proximal lesion SA together with AVR using a biological prosthesis. Atherosclerosis is the main cause of coronary artery ostial lesions.^4,5^ Secondary ostial lesions are associated with syphilitic vasculitis, rheumatoid arthritis, Takayasu’s arteritis, radiation therapy or injury following coronary intervention.^6,7^ In our presented case, radiation therapy-induced (due to breast cancer treatment) accelerated atherosclerotic disease is the most possible aetiology for the significant stenosis of both coronary ostia and the early calcification of the tricuspid AV.

Radiation‐induced cardiovascular disease (RICD) is an under-recognised clinical phenomenon. Coronary artery disease is the most common manifestation of RICD, with an incidence of up to 85%.^8^ The arterial narrowing seen with radiation is very proximal and usually involves the coronary ostia.^8^ As shown by a retrospective analysis^9^ of patients who underwent CABG after thoracic radiation, arterial conduits can be fibrotic, scarred, or stenosed in most cases, which is why SA could be the treatment of choice for those patients as they often have coronary ostium stenosis. SA was selected in our case to ensure full myocardial revascularisation due to the poor quality arterial conduits after radiation therapy (the radial artery graft was rejected due to an abnormal Allen test (peripheral vascular disease)) and extensive varicose veins of lower extremities.

The AV is usually the closest to the radiation field, increasing the risk of subsequent disease.^10^ Transcatheter AVR seems to be a promising alternative for the treatment of radiation‐induced AS, with lower mortality than surgical AVR,^11,12^ especially in patients at high risk of surgery, but it was not possible in our case due to the complex pathology (coronary ostium stenosis together with AS).

The biggest dataset about short- and long-term outcomes after SA came from a systematic literature review,^3^ which identified 478 patients undergoing isolated LMCA ostium stenosis SA. According to their data,^3^ the 30-days mortality was 1.7% and cardiac specific mortality was 3.3% at the last follow-up. It should be added that 92.4% of the reported cases showed complete angiographic patency at the last follow-up (after 54 months).^3^

A variety of patch materials can be used during SA, including the pericardium, saphenous vein and internal mammary and pulmonary arteries.^13–16^ Autologous pericardium is the most commonly used patch material and was also used in our case. It is widely available and allows the preservation of future conduit material, but lacks fibrinolytic properties that could theoretically promote calcification and restenosis. Data of previous small studies are quite contradictory, so a larger and randomised study could answer the question of which patch is the best.

Antiplatelets and anticoagulants reduce the risk of early occlusion and promote long-term graft patency following conventional CABG. In the largest series (*n* = 91), Maureira et al.^15^ reported a repeat myocardial revascularisation rate of 12 ± 6.8% at 5 years after SA, despite early anticoagulation with both aspirin and 3 months of warfarin. Comparable results have been reported with less aggressive strategies utilising aspirin alone.^16^ In our case, the patient was treated with a combination of an anticoagulant and antiplatelet for the first 3 months, considering that a biological AV prosthesis was implanted and SA of both ostia was performed. After 3 months, the treatment was continued with aspirin and clopidogrel, leaving aspirin for the continuous long-term treatment.

## Conclusions

Surgical coronary angioplasty offers an alternative to conventional coronary artery bypass grafting in isolated coronary ostial lesions and is advantageous in restoring more physiological myocardial perfusion, especially in those cases where conduits are suspected to be fibrotic, scarred or stenosed or if there is the need to preserve conduits for future myocardial revascularisation.
